# Depressive mixed state and anxious distress as risk factors for suicidal behavior during major depressive episodes

**DOI:** 10.1038/s41598-025-92437-3

**Published:** 2025-04-07

**Authors:** Kazuki Ota, Hotaka Shinzato, Naoaki Otsuka, Yu Zamami, Kazuhiro Kurihara, Kunihiro Futenma, Tsuyoshi Kondo, Yoshikazu Takaesu

**Affiliations:** Department of Neuropsychiatry, Graduate School of Medicine, University of the Ryukyus, 207 Uehara, Nishihara, Okinawa 903-0215 Japan

**Keywords:** Anxious distress, Depressive mixed state, DMX-8, Major depressive episodes, Suicidal behavior, Emotion, Social neuroscience

## Abstract

Accurately assessing and predicting suicidal behavior in patients with depression are challenging for researchers and clinicians. We examined various risk factors for suicidal behavior during major depressive episodes (MDE), especially focusing on depressive mixed state (DMX) and anxious distress (AD). We recruited 187 patients with MDE and divided them into two groups—with and without suicidal behavior—defined as the cut-off score of 1 or more on the suicidal behavior sub-item in the quick inventory of depressive symptomatology-self report. The presence of DMX was defined as a total score of 13 or more on the self-administered 8-item questionnaire for DMX. We used multivariate logistic regression analysis with the presence or absence of suicidal behavior as a dependent variable for investigating factors associated with suicidal behavior. The with suicidal behavior group was younger and indicated a greater proportion of past suicide attempts, AD, and DMX than the without suicidal behavior group. Logistic regression analysis revealed that AD (*P* = 0.020) and DMX (*P* = 0.018) were significantly associated with suicidal behavior. AD and DMX may promote suicidal behavior during MDE. These two psychopathological features should be carefully monitored and intensively treated for the prevention of suicide-related events.

## Introduction

Suicide is a serious global public health issue and is among the top twenty leading causes of death worldwide. Approximately 800,000 people die from suicide every year^1^. Additionally, previous reports have indicated a significant relationship between suicide and major depressive disorder (MDD)^2–4^. Depression and suicidal behavior, if unaddressed for a long time, can result in poor life quality and even lead to death^5^.

Mood disorders are considered heterogeneous conditions, and it has been suggested that they may encompass several subtypes. Various reports based on the inflammation hypothesis have been published, such as the association between the neutrophil-to-lymphocyte ratio and bipolar disorder^6^. Our previous research has also reported an association between depressive mixed states (DMX) and neurotrophic factors^7^. However, no inflammatory marker currently has solid evidence to clinically identify subtypes. Additionally, studies attempting to classify subtypes based on specific neural mechanisms have been conducted. Research using functional MRI has produced reproducible findings, such as the involvement of the default mode network^8^. However, inconsistent results have also been reported, and these findings have not yet been applied to the classification of subtypes in clinical practice. Further research is needed in this area^8^.

In the DSM-5, specifiers for major depressive episodes have been defined as subcategories. These specifiers include those with mixed features, those with anxious features (AD), and those with melancholic features, among others. A recent study suggested that some specifiers during major depressive episodes (MDE) according to the DSM-5 might play an important role in affecting worsened clinical features and outcomes in patients with MDE^9^. Among specifiers during MDE, DMX and AD may need special caution concerning suicidal behavior according to a previous network analysis^10^. Additionally, In mood disorders, nonadherence to prescribed treatment regimens is a significant issue, leading to poor clinical outcomes such as increased relapse rates, hospitalizations, and suicide risk^11^. Factors such as substance use disorders, illness severity, and treatment-related side effects are primary predictors of nonadherence, particularly in patients with major depressive disorder^11^. Recognizing and addressing these predictors is essential for improving adherence and treatment success. Building upon previous research, we hypothesize that DMX and AD are independent predictors of suicidal behavior during MDE. A recent study has shown that depressive episodes with anxious distress share significant overlap with bipolarity, suggesting a common underlying neurobiological mechanism^10^. This supports our hypothesis that the heightened emotional reactivity and impulsivity associated with AD and DMX contribute to the increased risk of suicidality.

DMX has been consistently discussed in relation to suicidal behavior in previous studies^12–14^. Therefore, it is crucial for clinicians to identify DMX for risk assessments and prevention of suicide during MDE^14^. However, appropriately assessing DMX remains challenging for clinicians, due to the complex features of DMX characterized by the simultaneous existence of depressive and issues, manic components^15^. The DSM-5 defines DMX as “mixed features” with at least three typical hypomanic/manic symptoms during MDE^16^. However, the DSM-5 criteria exclude irritability, psychomotor agitation, and inner tension from the mixed features criteria, which have been suggested to be more important symptoms because they are much more frequently observed in DMX compared to typical hypomanic/manic symptoms^17^. Therefore, the DSM-5 diagnostic criteria might be too insensitive to fully assess real-world DMX in a clinical setting^18^. To address these issues, we developed the self-administered 12-item questionnaire for DMX (DMX-12)^19^ to assess DMX, from which we extracted the eight most effective items (DMX-8) for screening purposes, and clarified that the DMX-8 can distinguish not only mixed features^16^ but also DMX with considerable severity^20^. The DMX-8 consists of eight items: overreactivity, inner tension, racing/crowded thought, impulsivity, irritability, aggression, risk-taking behavior, and dysphoria^20^. Each item is scored on a 4-point Likert scale ranging from 0 to 3, with a total score of 24. Its external validity was tested in patients with DSM-5-defined mixed features and DMX, and it was reported that a score of 13 or higher effectively screens for both^20^.

Meanwhile, AD was introduced in DSM-5 as a specifier of MDD, emphasizing the clinical significance of anxiety in depressed patients^21^. The DSM-5 criterion for the AD specifier is the presence of at least: (1) feeling keyed up or tense, (2) feeling unusually restless, (3) difficulty concentrating because of worry, (4) fear that something awful might happen, and (5) feeling that the individual might lose control of himself or herself^16^. The AD specifier captures core symptoms of anxious emotions and cognitions. One study indicated that the AD specifier according to the DSM-5 is a reliable and valid measure, with significant discriminant and convergent validity^22^. Another study indicated that AD is likely associated with suicidal behavior^9^.

Although DMX assessed by the DMX-8 and AD defined by the DSM-5 are independent subcategories during MDE, these specifiers share some overlapping psychopathology such as psychic agitation, inner tension, and feeling keyed up or on edge^23^. Therefore, the aim of this study was to clarify whether DMX and AD were independently associated with suicidal behavior, and which psychopathology had a stronger impact on suicidal behavior in patients with MDE.

## Results

### Comparison of characteristics and clinical backgrounds

The patients’ average age was 43.5 ± 17.7 years, and 71 (38.0%) were male. The average age in the with-suicidal behavior group was significantly younger than in the without-suicidal behavior group (41.5 ± 17.3 vs. 48.3 ± 18.3, *P* = 0.019, Cohen’s d = 0.389). The rate of patients with past suicide attempts was significantly higher in the with-suicidal behavior group compared to the without-suicidal behavior group (34.8% vs. 13.7%, *P* = 0.006, Cramér’s V = 0.205). The rate of patients with AD was significantly higher in the with-suicidal behavior group compared to the without-suicidal behavior group (31.6% vs. 9.8%, *P* = 0.002, Cramér’s V = 0.222). The rate of patients with DMX was significantly higher in the with-suicidal behavior group compared to the without-suicidal behavior group (48.5% vs. 17.6%, *P* < 0.001, Cramér’s V = 0.281). No significant differences were observed in other factors (gender distribution, living environment, Educational attainment, marital status, drinking habits, family history of suicide, Physical comorbidities, Bipolar disorder diagnoses, comorbid ASD/ADHD, rapid cycling, psychotic features, melancholic features and mixed features) between the groups with and without suicidal behavior.

Additionally, the rate of patients with DMX in the with-AD group was significantly higher than in the without-AD group (78.4 vs. 46.4%, *P* < 0.001, Cramér’s V = 0.325).

### Comparison of the suicidal behavior sub-item of QIDS-SR-J score among four groups with and without DMX (+) and AD

**Fig. 1:**
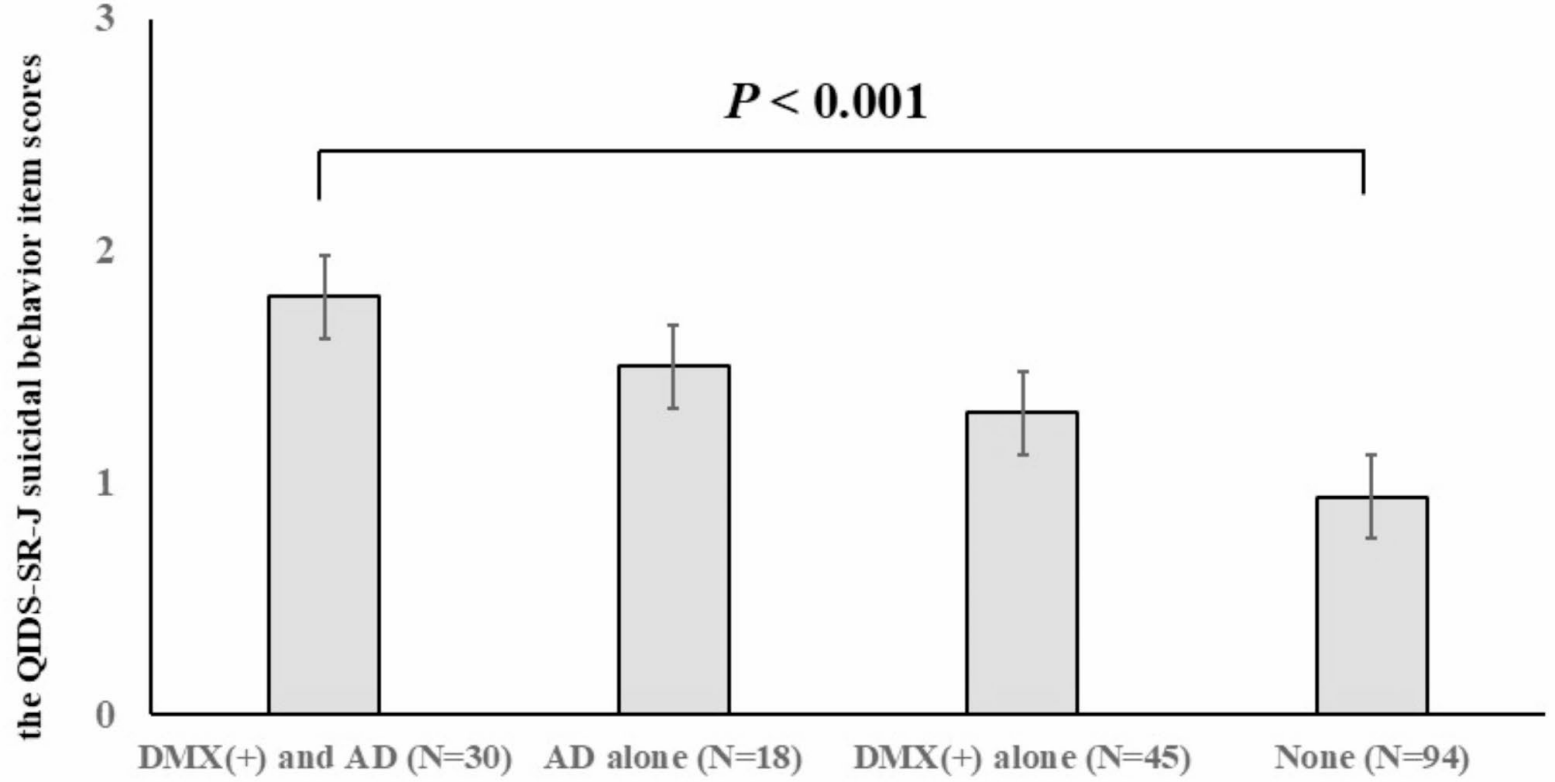
Item scores of suicidal behavior from QIDS-SR-J among four groups with and without DMX and AD. The vertical axis represents the QIDS-SR-J suicidal behavior item scores, while the horizontal axis categorizes the four groups based on the presence or absence of DMX and AD. *P* < 0.05 was considered a significant correlation. (*P* < 0.001: ANOVA followed by the Tukey test as a post-hoc analysis). *DMX* depressive mixed state, *AD* anxious distress, *QIDS-SR-J* the quick inventory of depressive symptomatology-self report-Japanese version.

One-way ANOVA revealed a significant difference in the suicidal behavior sub-item of the QIDS-SR-J score among groups with and without DMX (+) and AD. The groups with and without DMX (+) and AD were distributed as follows: with DMX (+) and AD (*n* = 30), AD alone (*n* = 18), DMX (+) alone (*n* = 45), and none (*n* = 94). Post-hoc analysis revealed that the suicidal behavior sub-item of the QIDS-SR-J score in the DMX (+) and AD group was significantly higher than the “none” group (1.83 ± 0.9 vs. 0.94 ± 1.0, *P* < 0.001), respectively (Fig. 1).

### Multivariate logistic regression analysis for risk factors with suicidal behavior

Univariate analysis of the clinical background revealed that younger age (odds ratio [OR] = 0.98, 95% confidence interval [CI] 0.96–0.99, *P* = 0.021), past suicide attempts (OR = 3.36, 95% CI 1.40–8.03, *P* = 0.007), AD (OR = 4.25, 95% CI 1.58–11.46, *P* = 0.004), and DMX (+) (OR 4.40, 95% CI 1.99–9.74, *P* < 0.001) were significantly associated with the suicidal behavior. After adjusting for confounding factors, multivariate logistic regression analysis revealed that AD (OR 3.58, 95% CI 1.22–10.5, *P* = 0.020) and DMX (+) (OR = 2.78, 95% CI 1.19–6.48, *P* = 0.018) were significantly associated with suicidal behavior (Table 1).

**Table 1:** Multivariate logistic regression analysis of plausible risk factors for suicidal behavior in patients during major depressive episodes.

Variables	Crude odds ratio (95% CI)	*P* value	Multivariate adjusted odds ratio (95% CI)	*P* value
Age	0.98 (0.96–0.99)	0.021	0.98 (0.96–1.00)	0.078
Sex	0.74 (0.39–1.43)	0.373		
Living alone	0.73 (0.31–1.73)	0.470		
College graduate	1.04 (0.49–2.22)	0.917		
Employed	1.52 (0.79–2.93)	0.207		
Married	0.65 (0.34–1.25)	0.192		
Habitual alcohol drinking	0.83 (0.34–2.05)	0.684		
Family history of suicide	6.26 (0.80–49.1)	0.081		
Past suicide attempts	3.36 (1.40–8.03)	0.007	2.09 (0.82–5.29)	0.121
Physical comorbidity	0.71 (0.37–1.37)	0.304		
Bipolar diagnosis	0.57 (0.26–1.24)	0.153		
Comorbid ASD/ADHD	0.88 (0.39-2.00)	0.757		
Rapid cycle of mood swing	3.74 (0.84–16.7)	0.085		
Anxious distress	4.25 (1.58–11.46)	0.004	3.58 (1.22–10.5)	0.020
Psychotic features	1.44 (0.45–4.56)	0.538		
Melancholic features	0.60 (0.21–1.73)	0.341		
Mixed features	2.71 (0.33–22.6)	0.356		
DMX (+)	1.15 (1.09–1.20)	< 0.001	2.78 (1.19–6.48)	0.018

Multivariate logistic regression analysis was used for this analysis. The table includes the odds ratio (OR), 95% confidence interval (CI), and p-value for each variable, highlighting the strength and significance of these associations.

*ASD* autism spectrum disorder, *ADHD* attention-deficit/hyperactivity disorder, *DMX* depressive mixed state.

## Discussion

The primary findings of this study revealed that DMX and AD are independent predictors of suicidal behavior during MDE. Previous research has dealt with various risk factors for suicidal behavior during MDE, such as psychiatric comorbidities including anxiety disorders, physical comorbidities, and a history of suicide. However, few studies have specifically focused on the relationship between suicidal behavior and specifiers of mood disorders, including mixed features and AD according to the DSM-5. To address this gap, this study comprehensively and simultaneously evaluated the effects of clinical backgrounds, diagnosis (bipolar and neurodevelopmental disorders), various specifiers (psychotic, melancholic, mixed features, and AD), and sub-categorical psychopathology (DMX) on suicidality. Consequently, only DMX and AD were independently associated with suicidality in patients with MDE.

Our previous study using the DSM-IV criteria identified atypical pervasive developmental disorder as a risk factor for suicide attempts with more lethal methods in adult patients with depression^24^. However, our present study using DSM-5 criteria failed to find a relationship between suicidal behavior and ASD. Plausible reasons for this discrepancy may be at least partly explained by the differences in the criteria used (patients with more atypical autism spectrum were included in the previous study using DSM-IV) and the range of suicidal behavior (broader suicidal behavior including suicidal ideation was dealt with in the present study) between the two studies.

This study has highlighted a close association between DMX and suicidal behavior, which is almost consistent with previous studies^25,26^. However, one recent systematic review of 16 cross-sectional studies indicated no difference in suicidal behavior between MDD with and without DMX^27^. As mentioned above, the results of previous studies investigating suicidal behavior concerning DMX were inconsistent, probably because of the heterogeneity in categorical criteria of DMX used in the studies^28^. This study revealed that DMX, but not mixed features, significantly affects suicidal behavior in patients with MDE (Table 1). To date, DMX has been diversely defined since Kraepelin proposed the concept of mixed states^29^. However, mixed features by the DSM-5 were deemed diagnostically specific but practically insensitive owing to the exclusion of nonspecific but prevalent symptoms for DMX^30,31^, leading to underdiagnosis of DMX^31–33^. This was also supported by our previous finding that the rate of mixed features by DSM-5 was only 4.2% while that of DMX was 22.6% using the same samples with MDE^18^.

AD has also been suggested to be associated with suicidal behavior in several previous studies^21,22^. A 2-year longitudinal cohort study compared clinical outcomes of MDD (severity and chronicity of the disease, functional impairment, presence of comorbid anxiety disorders, and prevalence of suicidal behavior) between MDD with and without AD^21^. The study concluded that MDD with AD generally exhibited worse clinical outcomes than MDD without AD^21^. Another study later confirmed that the presence of AD was closely associated with a greater prevalence of bipolarity, mixed features, and suicidal behavior during MDE^34^. Our cross-sectional study also supported that AD was an independent risk factor for suicidal behavior (Table 1). Furthermore, as illustrated in (Fig. 1), it should be noted that the coexistence of AD and DMX during MDE significantly increases the risk of suicidal behavior due to their additive or synergistic effects.

DMX and AD have been considered independent factors; however, they exhibit some shared psychopathology (psychic agitation, inner tension, and feeling keyed up or on edge) despite being different subcategories for depressive disorders^9^. Although such overlapped psychopathology comprises rather nonspecific manic/hypomanic features, these basal symptoms underlying mixed pathology might have a dynamic impact on severe outcomes of depression like treatment resistance and risk of suicidal behavior^34–36^. Although multivariate logistic regression analysis demonstrated that both DMX and AD were independent risk factors (Table 1), 40% of patients with DMX had AD and 62.5% of patients with AD had DMX in this study, suggesting a reciprocal affinity between DMX and AD. Furthermore, our study revealed that 30 out of 187 (16.0%) patients exhibited DMX and AD, further accelerating the risk of suicidal behavior (Fig. 1). Thus, the most attention should be paid to these cases of coexisting DMX and AD during MDE for the prevention of suicide.

This study had several limitations. First, the study was conducted using a single-center design with a relatively small sample size, which may have reduced the statistical power and generalizability of the findings. Second, the study’s cross-sectional design precludes any causal inferences between suicidality and DMX or AD. Longitudinal studies are needed to establish these relationships. Third, suicidality was assessed using the QIDS-SR-J, a self-report measure. While this tool is widely used and validated, reliance on self-report measures introduces the potential for response bias, including denial or underestimation of suicidality. Future studies should consider incorporating clinician-rated scales or objective assessments to mitigate these biases. In this study, the diagnoses were confirmed by two specialists. However, as structured interviews and IQ tests were not conducted, the possibility of bias in the evaluation cannot be ruled out.

In conclusion, our data indicated that DMX and AD are independent risk factors for suicidal behavior during MDE. In particular, clinicians need to pay the most attention to cases of coexisting DMX and AD, indicating the highest risk for suicidal behavior.

### Clinical recommendations

The findings of this study highlight the critical importance of identifying and addressing DMX and AD in clinical settings. Both DMX and AD were found to be independent predictors of suicidal behavior, emphasizing the need for routine screening during psychiatric evaluations. As noted by a recent study, the inclusion of DSM-5 specifiers such as anxious distress improves the precision of identifying high-risk patients^37^. Early detection of DMX and AD allows for targeted interventions, such as cognitive-behavioral therapy to address emotional dysregulation in DMX or pharmacological treatment for anxiety symptoms in AD. These measures may significantly reduce suicide risk and improve overall treatment outcomes.

## Methods

### Patients

This study was conducted at the University of the Ryukyus Hospital in Japan from June 2014 to December 2019. The sample size was calculated based on a medium effect size (Cohen’s d = 0.5), a significance level of 0.05, and a statistical power of 0.8, with a target of at least 128 participants. A total of 301 new consecutive outpatients with suspected MDE were presented to our outpatient clinic and diagnosed by two experienced psychiatrists according to the DSM-5 diagnostic criteria^16^. Two board-certified psychiatrists determined whether or not the patient met the DSM-5 diagnosis of a current depressive episode. The 298 patients whose native language was Japanese were included in the study, two of whom withdrew their consent during the study. Patients with substance-related/addictive disorders, neurocognitive disorders, or intellectual disabilities were excluded from the study. Two board-certified psychiatrists diagnosed all these comorbid conditions based on the DSM-5 criteria. Consequently, 190 patients (72 male and 118 female) were included in the analysis. Two board-certified psychiatrists diagnosed the patients with MDE according to the DSM-5 criteria, including 139 patients with MDD and 51 patients with bipolar and related disorders. The psychiatrists also diagnosed comorbid autism spectrum disorder (ASD), attention-deficit/hyperactivity disorder (ADHD), and specifiers for depressive disorders according to the DSM-5 criteria. Three participants were excluded from the analysis due to incomplete responses in the questionnaire.

### Clinical assessment

This study utilized two self-administered scales: the QIDS-SR-J and the DMX-8. Suicidal behavior was evaluated using the Quick Inventory of Depressive Symptomatology-Self Report^38^ Japanese version (QIDS-SR-J). The QIDS-SR-J is a validated tool for assessing depressive symptoms^38^. In this study, the suicidality subscale was used, which ranges from 0 to 3, with higher scores indicating a greater risk for suicidal behavior. Patients were divided into two groups—with and without suicidal behavior —based on the cut-off score (1 or more) of the suicidal behavior sub-item in the QIDS-SR-J. DMX was evaluated by the DMX-8^34^. The 8 items include over-reactivity, inner tension, racing thoughts, impulsivity, irritability, aggression, risk-taking behavior, and dysphoria. Each item on the DMX-8 is scored on a 4-point scale based on the frequency of each symptom (0: never; 1: only occasionally; 2: often; 3: almost always) during the latest 1-week period of MDD. Categorical screening as DMX was determined by a cutoff score of ≥ 13 for the eight DMX-specific symptoms from DMX-8 (over-reactivity, inner tension, racing thoughts, impulsivity, irritability, aggression, risk-taking behavior, and dysphoria)^18^. Patients meeting this criterion were designated as DMX (+).

### Statistical analyses

The Student’s t-test was used for the comparison of continuous variables (age) between patients with and without suicidal behavior. The chi-squared test was used for the comparison of categorical variables: sex; living alone; educational background; employment status; marital status; habitual alcohol consumption; family history of suicide; past suicide attempts; physical comorbidity; comorbidity with neurodevelopment disorders including ASD and ADHD; DSM-5 diagnostic specifiers of rapid cycle mood swing of bipolar and related disorders, AD, psychotic symptoms, melancholic disposition, and mixed features; diagnosis of mood disorders; and DMX (+) between the two groups. One-way analysis of variance (ANOVA), followed by Turkey’s post-hoc test, was used for the comparison of the suicidal behavior sub-item of the QIDS-SR-J score among four groups with and without DMX (+) and AD. To investigate the factors associated with suicidal behavior, we used multivariate logistic regression analysis with the presence or absence of suicidal behavior as a dependent variable and the following as independent variables: age, proportion of patients with past suicide attempts, rate of AD, and rate of DMX (+). Potential confounders, such as age and past suicide attempts, were considered during the analysis. These factors were included as covariates in the logistic regression models to control for their potential influence on the relationship between DMX, AD, and suicidal behavior. By adjusting for these confounders, we aimed to ensure that the observed associations represent the independent effects of DMX and AD on suicidal behavior. Our study used forced entry methods as the logistic regression model, focusing on variables of clinical significance. In the series of logistic regression analyses, variables that exhibited significant association in univariate analyses were put into a multivariate model. The logistic regression model was evaluated for its goodness-of-fit and explanatory power. The Nagelkerke R² was 0.206, indicating moderate explanatory power. The model’s goodness-of-fit was assessed using the Hosmer-Lemeshow test, which yielded a χ² value of 3.952 with 8 degrees of freedom (*p* = 0.861), suggesting a good fit to the data. SPSS version 27 software for Windows (SPSS Inc., Chicago) was used for all statistical analyses. A two-tailed P value less than 0.05 was considered to indicate a statistically significant difference.

### Research ethics

Written informed consent was obtained from all participants involved in the study. For patients under the age of 18 years, written informed consent was obtained not only from patients themselves but also from their parents. This study protocol was conducted following the Declaration of Helsinki and was approved by the Ethics Committee at the University of Ryukyus.

## Data Availability

The data from this study are not publicly available because the disclosure of individual data was not included in the research protocol, and consent for public data sharing was not obtained from the participants. However, the data are available from the corresponding author upon reasonable request.

## References

[1] World Health Organization. Suicide in the world: global health estimates. http://apps.who.int/bookorders (World Health Organization, 2019)

[2] Nawaz, H., Shah, I. & Ali, S. The amygdala connectivity with depression and suicide ideation with suicide behavior: a meta-analysis of structural MRI, resting-state fMRI and task fMRI. *Prog Neuro-Psychopharmacol. Biol. Psychiatr.* **124**, 110736 (2023). 10.1016/j.pnpbp.2023.110736 36842608

[3] Paljärvi, T. et al. Psychotic depression and deaths due to suicide. *J. Affect. Disord*. **321**, 28–32 (2023). 36280195 10.1016/j.jad.2022.10.035

[4] Sun, F. K. et al. The effects of logotherapy on meaning in life, depression, hopelessness, and suicidal ideation, in patients with depression: an intervention study. *Perspect. Psychiatr. Care* **58**, 1891–1899 (2022). 34923643 10.1111/ppc.13003

[5] Dold, M. et al. Major depression and the degree of suicidality: results of the European group for the study of resistant depression (GSRd). *Int. J. Neuropsychopharmacol.* **21**, 539–549 (2018). 29860382 10.1093/ijnp/pyy009 PMC6007240

[6] Fusar-Poli, L., Natale, A., Amerio, A., Cimpoesu, P., Grimaldi Filioli, P., Aguglia,E., Aguglia, A. Neutrophil-to-lymphocyte, platelet-to-lymphocyte and monocyte-to-lymphocyte ratio in bipolar disorder. *Brain Sci.* **11** (1), 58. (2021). 10.3390/brainsci11010058 PMC7825034 33418881

[7] Otsuka, N. et al. Elevated brain-derived neurotrophic factor levels during depressive mixed states. *Psychiatr. Investig.* **20** (11), 1027–1033 (2023). 37997330 10.30773/pi.2023.0104 PMC10678153

[8] Pilmeyer, J. et al. Functional MRI in major depressive disorder: A review of findings, limitations, and future prospects. *J. Neuroimaging* **32** (4), 582–595 (2022). 35598083 10.1111/jon.13011 PMC9540243

[9] Gaspersz, R. et al. Longitudinal predictive validity of the DSM-5 anxious distress specifier for clinical outcomes in a large cohort of patients with major depressive disorder. *J. Clin. Psychiatr.* **78**, 207–213 (2017). 27035515 10.4088/JCP.15m10221

[10] Tundo, A. et al. The relationship between depression with anxious distress DSM-5 specifier and mixed depression: a network analysis. *CNS Spectr.* **26**, 251–257 (2021). 32122436 10.1017/S1092852920000085

[11] Pompili, M. et al. Mood disorders medications: predictors of nonadherence–review of the current literature. *Expert Rev. Neurother.* **13**, 809–825 (2013). 23898852 10.1586/14737175.2013.811976

[12] Baldessarini, R. J., Vázquez, G. H. & Tondo, L. Bipolar depression: a major unsolved challenge. *Int. J. Bipolar. Disord.* **8**, 1 (2020). 10.1186/s40345-019-0160-1 PMC6943098 31903509

[13] Swann, A. C. Mixed features: evolution of the concept, past and current definitions, and future prospects. *CNS Spectr.* **22**, 161–169 (2017). 28264741 10.1017/S1092852916000882

[14] Persons, J. E. et al. Mixed state and suicide: is the effect of mixed state on suicidal behavior more than the sum of its parts? *Bipolar Disord.* **20**, 35–41 (2018). 28833953 10.1111/bdi.12538 PMC6237077

[15] Cervone, A. et al. Mixed States: diagnosis, assessment and diagnostic stability. *Psychiatr. Danub* **34** (8), 38–41 (2022). 36170699

[16] American Psychiatric Association. *Diagnostic and Statistical Manual of Mental Disorders* 5th edn (American Psychiatric Assoc., 2013).

[17] Shim, I. H., Bae, D. S. & Bahk, W. M. Anxiety or agitation in mood disorder with mixed features: a review with a focus on validity as a dimensional criterion. *Ann. Clin. Psychiatr.* **28**, 213–220 (2016). 27490837

[18] Verdolini, N. et al. The state of the art of the DSM-5 with mixed features specifier. *Sci. World J.* 1–7 (2015). 10.1155/2015/757258 PMC4562096 26380368

[19] Shinzato, H., Koda, M., Nakamura, A. & Kondo, T. Development of the 12-item questionnaire for quantitative assessment of depressive mixed state (DMX-12). *Neuropsychiatr. Dis. Treat.* **15**, 1983–1991 (2019). 31406462 10.2147/NDT.S215478 PMC6642622

[20] Shinzato, H., Zamami, Y. & Kondo, T. The 12-item self-rating questionnaire for depressive mixed state (DMX-12) for screening of mixed depression and mixed features. *Brain Sci.* **10**, 678 (2020). 32992474 10.3390/brainsci10100678 PMC7601672

[21] Zimmerman, M. et al. Validity of the DSM-5 anxious distress specifier for major depressive disorder. *Depress. Anxiety* **36**, 31–38 (2019). 30311733 10.1002/da.22837

[22] Gaspersz, R. et al. Anxious distress predicts subsequent treatment outcome and side effects in depressed patients starting antidepressant treatment. *J. Psychiatr. Res.* **84**, 41–48 (2017). 27693981 10.1016/j.jpsychires.2016.09.018

[23] Tundo, A. et al. Is there a relationship between depression with anxious distress DSM-5 specifier and bipolarity? A multicenter cohort study on patients with unipolar, bipolar I and II disorders. *J. Affect. Disord.* **245**, 819–826 (2019). 30699865 10.1016/j.jad.2018.11.024

[24] Takara, K. & Kondo, T. Comorbid atypical autistic traits as a potential risk factor for suicide attempts among adult depressed patients: a case-control study. *Ann. Gen. Psychiatr.* **13**, 33 (2014). 25328535 10.1186/s12991-014-0033-z PMC4201698

[25] Takeshima, M. Early recognition and appropriate pharmacotherapy for mixed depression: the key to resolving complex or treatment-refractory clinical cases. *Clin. Neuropsychopharmacol. Ther.* **10**, 10–17 (2019).

[26] Tondo, L. & Baldessarini, R. J. Antisuicidal effects in mood disorders: are they unique to lithium? *Pharmacopsychiatry* **51**, 177–188 (2018). 29672801 10.1055/a-0596-7853

[27] Lage, R. R., Santana, C. M. T., Nardi, A. E. & Cheniaux, E. Mixed States and suicidal behavior: a systematic review. *Trends Psychiatr. Psychother.* **41**, 191–200 (2019). 31291413 10.1590/2237-6089-2018-0042

[28] Koukopoulos, A., Sani, G. & Ghaemi, S. N. Mixed features of depression: why DSM-5 is wrong (and so was DSM-IV). *Br. J. Psychiatr.* **203**, 3–5 (2013). 23818531 10.1192/bjp.bp.112.124404

[29] Malhi, G. S. et al. Are manic symptoms that ‘dip’ into depression the essence of mixed features? *J. Affect. Disord.* **192**, 104–108 (2016). 26717522 10.1016/j.jad.2015.12.009

[30] Edinoff, A. N. et al. Brexpiprazole for the treatment of schizophrenia and major depressive disorder: a comprehensive review of Pharmacological considerations in clinical practice. *Psychopharmacol. Bull.* **51**, 69–95 (2021). 34092824 10.64719/pb.4399 PMC8146559

[31] Takeshima, M. Anxious distress in monopolar and bipolar depression: clinical characteristics and relation with mixed depression in Japan. *Psychiatr. Clin. Neurosci.* **72**, 456–457 (2018). 29652106 10.1111/pcn.12660

[32] Stahl, S. M. et al. Guidelines for the recognition and management of mixed depression. *CNS Spectr.* **22**, 203–219 (2017). 28421980 10.1017/S1092852917000165

[33] Zamami, Y. et al. Prevalence and profile of depressive mixed state in patients with autism spectrum disorder. *Psychiatr. Res.* **300**, 113932 (2021). 33887519 10.1016/j.psychres.2021.113932

[34] Sugawara, H. et al. Association between anxious distress in a major depressive episode and bipolarity. *Neuropsychiatr. Dis. Treat.* **15**, 267–270 (2019). 30697051 10.2147/NDT.S188947 PMC6339637

[35] Smith, K. A. & Cipriani, A. Lithium and suicide in mood disorders: updated meta-review of the scientific literature. *Bipolar Disord.* **19**, 575–586 (2017). 28895269 10.1111/bdi.12543

[36] Brown, T. M., DiBenedetti, D. B., Danchenko, N., Weiller, E. & Fava, M. Symptoms of anxiety and irritability in patients with major depressive disorder. *J. Depress. Anxiety* **5**, 237 (2016).

[37] Aoki, Y. et al. A psychometric analysis of the Japanese version of the clinically useful depression outcome scale supplemented with questions for the DSM-5 anxious distress specifier (CUDOS‐A). *Neuropsychopharmacol. Rep.* **44**, 526–533 (2024). 38838706 10.1002/npr2.12432 PMC11544442

[38] Rush, A. J. et al. The 16-Item quick inventory of depressive symptomatology (QIDS), clinician rating (QIDS-C), and self-report (QIDS-SR): a psychometric evaluation in patients with chronic major depression. *Biol. Psychiatr.* **54**, 573–583 (2003). 12946886 10.1016/s0006-3223(02)01866-8

